# The Role of Pupillometry in the Assessment of Pain in Children Under General Anesthesia: A Prospective Single-Blinded Observational Study

**DOI:** 10.7759/cureus.43894

**Published:** 2023-08-22

**Authors:** Akrity Singh, Akhileshwar, Nitin Kumar, Ranjeet Rana De, Raj Bahadur, Saurav Shekhar

**Affiliations:** 1 Department of Trauma and Emergency Medicine, Indira Gandhi Institute of Medical Sciences, Patna, IND

**Keywords:** surgical stimulus, opioids, pupillary reflex dilatation, nociception, pupillometry

## Abstract

Background and objective

The management and treatment of nociception remain one of the major challenges in anesthesiology, and hemodynamic variations may occur due to inadequate analgesia, which at times can be injurious. Pupillometry is a new noninvasive tool to assess nociception during anesthesia. The amount of pupillary reflex dilation (PRD) is directly proportional to the intensity of nociceptive stimuli and inversely proportional to the opioid dosage. This study aimed to assess the use of pupillometry as reflex pupillary dilatation in response to surgical stimulus in children under general anesthesia and to guide intraoperative opioid consumption.

Materials and methods

After obtaining approval from the institutional ethics committee and written consent from parents, children with an American Society of Anesthesiology (ASA) classification of I and II and aged 2-12 years who were undergoing surgery under general anesthesia were enrolled in this prospective randomized observational study. General anesthesia was standardized with propofol, sevoflurane, and O_2 _and N_2_O (50:50%), and fentanyl administration was guided by pupil diameter changes. The primary outcome was to measure pupillary dilatation in response to pain and fentanyl administration guided by it.

Results

A total of 72 patients were included in the study. The mean pupil diameter significantly increased after surgical stimulus from 1.37 ±0.87 to 2.40 ±1.95 mm (p<0.001). The heart rate (116.2 ±12.25 to 118.50 ±8.20 beats/minute, p=0.18) and systolic BP (114.60 ±17.73 to 118.50 ±12.25 mmHg, p=0.12) did not change significantly on stimulus. The mean fentanyl consumption was 2.4 ug/kg and the side effects were not remarkable.

Conclusion

Based on our findings, pain has a significant influence on the pupil dilatation reflex in anesthetized children, and opioid administration based on pupil diameter can be valuable in clinical settings. We recommend the use of pupillometry as a pain index in children undergoing surgery under general anesthesia, and it can be a beneficial tool for assessing intraoperative pain. Newer techniques and developments are required in this field.

## Introduction

The evaluation and measurement of nociception are one of the major concerns among anaesthesiologists during and after surgery. Nociception refers to the neural encoding of noxious stimulation, while pain is a subjective feeling of tissue injury or trauma. The noxious stimuli of the surgical trauma generated due to mechanical stress, pressure, or inflammation activate nociception in neurons, reaching the cerebral cortex even under general anesthesia through A-delta and C fibers. The hemodynamic variations result from inadequate analgesia intraoperatively and postoperatively, which can be injurious. The frequent administration of opioids is associated with side effects like nausea, vomiting, pruritus, slowed breathing, urinary retention, etc. Thus, it is important to calculate the minimum effective dose of intraoperative opioids or other analgesics required for an individual.

Hemodynamic parameters such as heart rate or blood pressure variations are commonly used to monitor analgesia intraoperatively. Many noninvasive devices have been developed in the past few years, with newer techniques, applications, and parameters, to check the balance between intraoperative algesia and analgesia. The dose of opioids should be individualized and effective to avoid overuse and underuse. Pupillometry is one such device and a reliable tool to monitor nociception. The pupillary diameter increases in response to painful stimuli. This phenomenon is called pupillary reflex dilation (PRD) and is observed in both conscious and anesthetized patients. The reflex dilation of the pupil in response to pain is correlated with the intensity of nociceptive stimuli and to the dose of administered opioids [1]. During surgery, pupillary diameter is thought to be a dynamic function of the intensity of surgical stimuli and opioid dosage. The sympathetic and parasympathetic divisions of the autonomic nervous system supply innervation to the smooth muscle of the iris and pupil size determination is the outcome of their opposite action. During general anesthesia, the sympathetic activity is depressed by the use of sedatives whereby the parasympathetic system activation is through the Edinger-Westphal (EW) nucleus, which results in miosis. In patients who are awake, PRD is due to the stimulation of the sympathetic pathway with a dilatation response [2]. In patients under anesthesia, the surgical painful stimulus inhibits the EW nucleus, leading to passive sphincter relaxation and reflex dilation. Opioids cause miosis by inhibiting the EW nucleus. Moreover, opioids' suppression of pupillary dilation is dose-related.

In light of this, we performed this study to assess pain by pupillometry in children under general anesthesia and opioid administration, to address the scarcity of data in the literature on the subject.

## Materials and methods

This study was conducted after obtaining ethical clearance from the Institutional Ethics Committee, IGIMS, Patna, and registering the trial with Clinical Trials Registry (CTRI/2022/02/040112). This was a prospective observational single-blinded study of children in the age group of 2-12 years of both sexes with the American Society of Anaesthesiologists (ASA) physical status I and II who were scheduled to undergo surgeries for lower abdominal or urological issues and upper and lower limbs under general anesthesia. The pupil measurements were taken by another anesthetist who was unaware of the anesthetic agents delivered and procedures performed (blinded).

The exclusion criteria were as follows: children whose parents declined to enroll in the study; children with a history of hypersensitivity to drugs, or use of anticholinergic drugs, opioids, and antiemetics recently; the presence of eye disease or any viral illness; a history of cardiovascular disease or anomaly; and those with a duration of surgery of more than two hours. Preanesthetic evaluation of all the patients was performed before admitting them to the ward.

**Figure 1:**
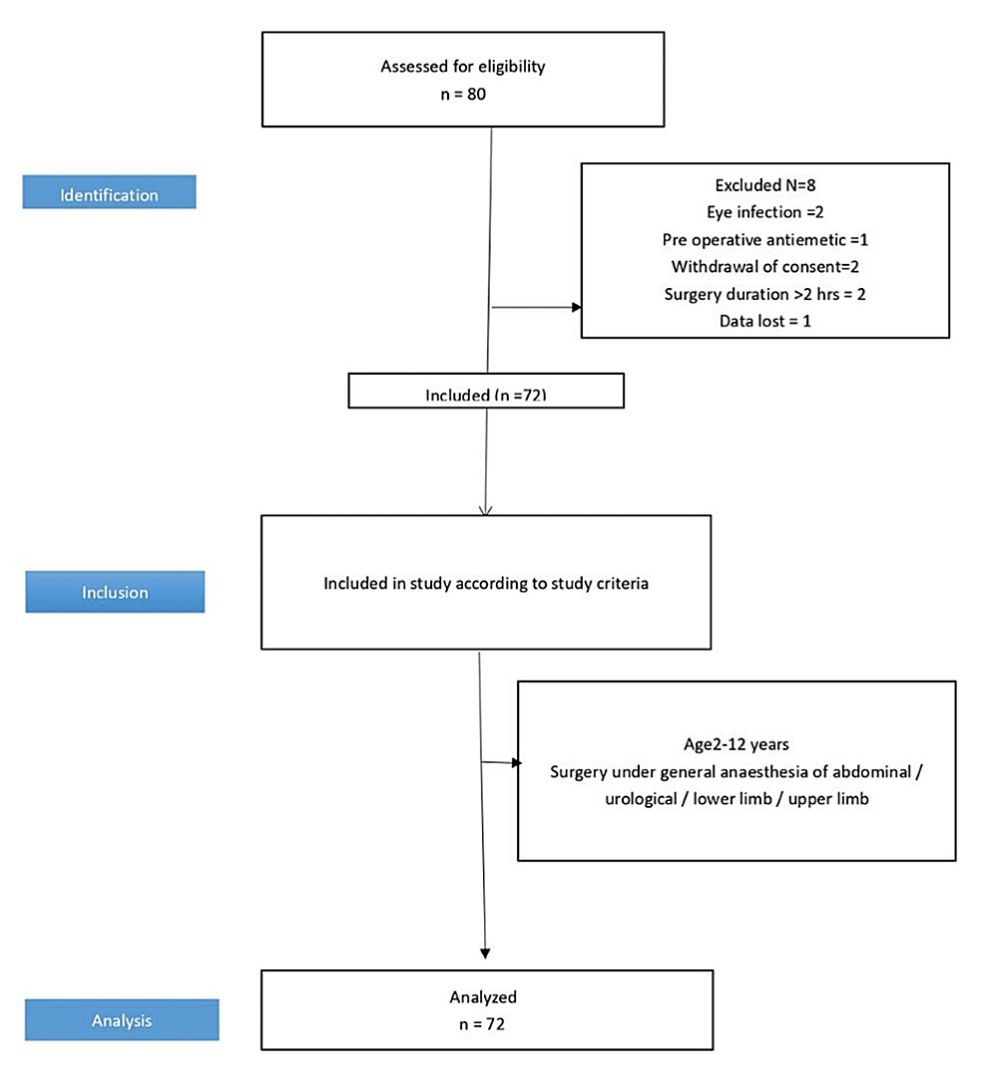
STROBE diagram STROBE: Strengthening the Reporting of Observational Studies in Epidemiology

Out of 80 patients assessed for eligibility in the study, 72 children were included in the final analysis and eight were excluded due to several reasons, as shown in the Strengthening the Reporting of Observational Studies in Epidemiology (STROBE) diagram (Figure 1).

**Figure 2:**
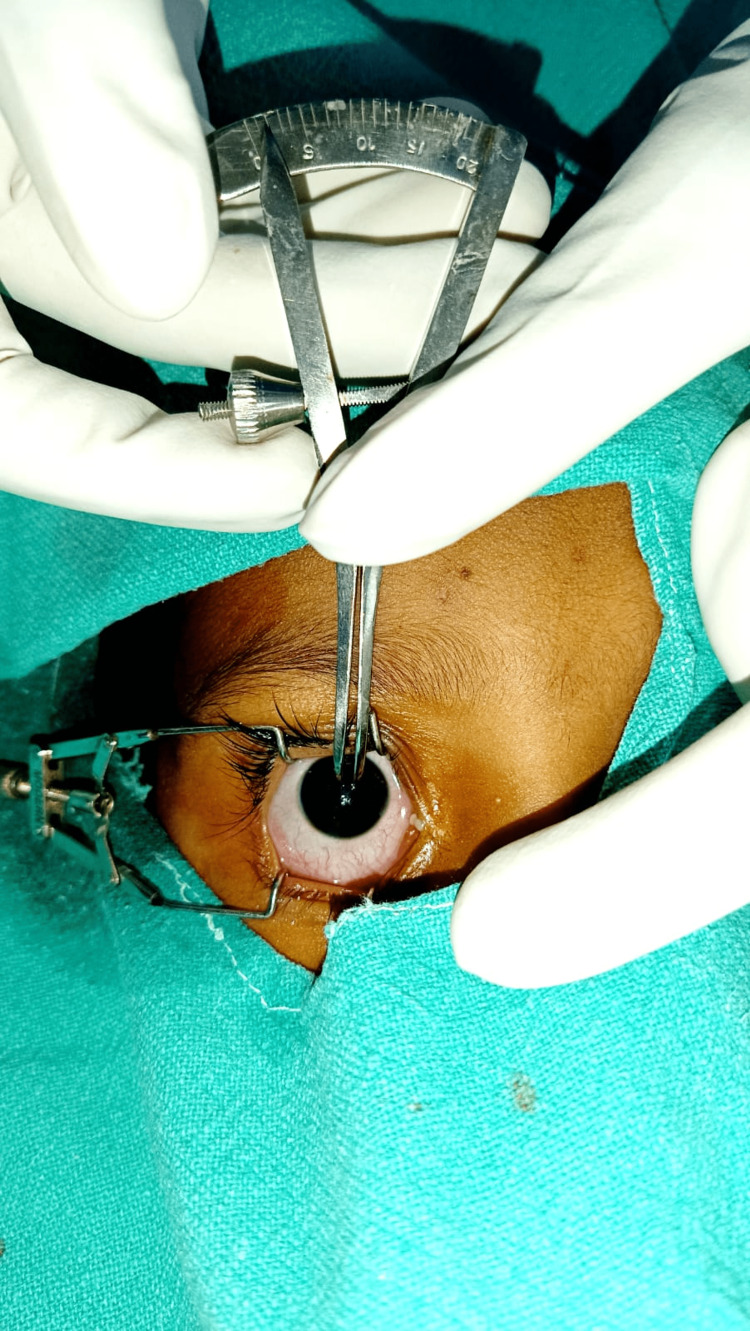
The measurement of pupil size by vernier caliper

On arrival at the operation theatre, routine monitoring in the form of electrocardiography (ECG), noninvasive arterial pressure (NIBP), pulse oximetry (SPO_2_), and respiration were instituted, and baseline values were noted. Intravenous access was established with a 20/22 G intravenous catheter on the dorsum of the nonoperative hand. The baseline eye examination was done to assess normal light reflex and rule out any pathology during the preanesthetic check-up. The pupil size was measured by pupillometer/digital vernier caliper after opening the eye with lid retractor (Figure 2) at baseline (under sedation, T0), at one minute after induction (T1), during skin incision (T2), after opioid use (T3), and before skin closure (T4) in the same eye, and other eye was covered. The caliper never touched the pupil.

No antiemetics were administered in the preoperative period and hypercarbia was avoided by keeping ETCO_2_ below 45 mmHg.

After taking written informed consent from the parent or legal guardian of the child, the patient was taken to the operation theatre. Induction was done with Inj. propofol 2 mg/kg of body weight and Inj. atracurium 0.5 mg/kg of body weight. and oxygen 100%. Hypnosis was maintained with sevoflurane [minimum alveolar concentration (MAC): 1.5], 50% oxygen, and 50% nitrous oxide, and ETCO_2_ was kept between 32 and 40 mmHg. Intraoperative monitoring of NIBP, respiratory rate, heart rate, ETCO_2_, and SpO_2_ was done, and IV fluids were given as per protocol. Intraoperative analgesia was administered in the form of fentanyl bolus 1 ug/kg titrated to variations and changes in the pupil size recorded. After the completion of the surgery, the neuromuscular blockade was reversed with Inj. neostigmine and glycopyrrolate combination and Inj. ondansetron 0.1 mg/kg was given before reversal. An antibiotic (ofloxacin eye drop) was instilled in both eyes after the measurement was over. Vitals were recorded during the recovery period and the patient was observed for 30 minutes after surgery in the postoperative area. Any adverse effects were noted and treated symptomatically. The patients were followed up in the postoperative period and in the ward till discharge.

The size of the pupil was recorded at different intervals [baseline under sedation (T0), at one minute after induction (T1), during skin incision (T2), after opioid use (T3), and before skin closure (T4)], as mentioned above, in ambient light and not in bright light by separating the surgical field and head area with a screen. The other eye remained covered to prevent contralateral light reflex, and stimulation was stopped when pupil dilatation was more than 13% of the baseline. Inj. fentanyl 1 ug/kg was then administered, and the next measurement was recorded after two minutes.

Sample size and statistical analysis

Continuous variables were examined for normality using the Shapiro-Wilk test and were reported as mean (SD) or median (IQR) where appropriate. Normally distributed and non-normally distributed variables were analyzed using a one-way analysis of variance (ANOVA) or the Kruskal-Wallis test, respectively. Categorical data were analyzed using the Chi-squared test. A p-value <0.05 was considered statistically significant for all comparisons. Data were analyzed using IBM SPSS Statistics for Windows (IBM Corp., Armonk, NY).

## Results

Data related to 72 children who underwent surgeries for lower abdominal or urological issues and upper and lower limbs under general anesthesia were collected. The demographics of children are presented in Table 1. There was no significant difference in terms of gender and ASA status of patients included in the study. The pupil diameter was measured at different time intervals along with BP and heart rate (Table 2). The change in heart rate (116.2 ±12.25 to 118.50 ±8.20 beats/minute, p=0.18) and systolic BP (114.60 ±17.73 to 118.50 ±12.25 mmHg, p=0.12) was not significant on surgical stimulus. However, pupil diameter significantly increased after surgical stimulus from 1.37 ±0.87 to 2.40 ±1.95 mm (p<0.001) (Table 3). Also, after the administration of opioids, the pupil diameter again decreased, which suggests that pain causes pupil dilatation and the administration of opioids causes miosis. Thus, pupil diameter measurement can be a useful guide to opioid requirements. The mean fentanyl consumption was 2.4 ug/kg (Table 4), and the side effects were not remarkable (Table 5).

**Table 1:** Patient demographics ASA: the American Society of Anesthesiologists; SD: standard deviation

Variable	Values
Age, years, mean ±SD	5 ±3
Weight, kg, mean ±SD	20 ±10
Length, cm, mean ±SD	102 ±10
Sex, n	38 M/34 F
ASA I, n (%)	39 (54%)
ASA II, n (%)	33 (46%)

**Table 2:** Pupil diameter measured at different time intervals and vitals recorded HR: heart rate; SBP: systolic blood pressure; SD: standard deviation

Time interval	Pupil size, mm, mean ±SD	HR, beats/minute, mean ±SD	SBP, mmHg, mean ±SD
Baseline (T0)	3.57 ±1.09	122 ±12.59	120.55 ±18.47
1 minute after induction (T1)	1.37 ±0.87	116.2 ±12.25	114.60 ±17.73
At skin incision (T2)	2.4 ±1.95	118.50 ±8.20	118.50 ±12.25
2 minutes after opioid use (T3)	1.1 ±0.23	115.80 ±8.22	116.20 ±16.36
Before skin closure (T4)	2.72 ±1.15	113.64 ±16.94	109.09 ±14.92

**Table 3:** Comparison of pupil size, heart rate, and systolic BP at different time intervals HR: heart rate; SBP: systolic blood pressure; SD: standard deviation

Parameters	Base (T0), mean ±SD	Pre-stimulus (T1), mean ±SD	P-value	Pre-stimulus (T1), mean ±SD	Stimulus (T2), mean ±SD	P-value	Stimulus (T2), mean ±SD	After opioid use (T3), mean ±SD	P-value
HR, beats/minute	122 ±12.59	116.2 ±12.25	0.48	116.2 ±12.25	118.50 ±8.20	0.18	118.50 ±8.20	115.80 ±8.22	0.05
SBP, mmHg	120.55 ±18.47	114.60 ±17.73	0.48	114.60 ±17.73	118.50 ±12.25	0.12	118.50 ±12.25	116.20 ±16.36	0.34
Pupil size, mm	3.57 ±1.09	1.37 ±0.87	0.21	1.37 ±0.87	2.40 ±1.95	<0.001	2.40 ±1.95	1.1 ±0.23	<0.001

**Table 4:** Mean fentanyl requirement and duration of anesthesia

Mean fentanyl requirement	Duration of anesthesia
2.4 ug/kg	1.4 ±0.5 hours

**Table 5:** Incidence of side effects

Side effects	Incidence, n (%)
Postop analgesic requirement	12 (16.6%)
Nausea/vomiting	10 (13.8%)
Pruritus	4 (5.5%)

## Discussion

The assessment and treatment of pain in children are difficult to evaluate as compared to adults, which often results in either undertreatment or overtreatment. The treatment of pain in children and adolescents is now focused on an interdisciplinary approach, with an equal emphasis on therapeutic, pharmacological, psychosocial, and behavioral aspects. The early evaluation and control of pain symptoms have improved with the emergence of newer techniques. The balance between nociception due to surgical stress and antinociception induced by anesthesia has an impact on the patient's homeostasis and postoperative recovery. Various nociceptive monitors are available to measure pain during anesthesia, such as analgesia nociception index (ANI), surgical pleth index (SPI), enhanced recovery after surgery (ERAS) nociception level (NOL), and pupillary pain index (PPI). Our study evaluated the use of a pupillometer as an alternative to other variables like heart rate and blood pressure in analyzing pain and depth of sedation during general anesthesia in children and in guiding intraoperative fentanyl requirements.

In our study, we found that surgical stimulus causes reflex pupillary dilatation under anesthesia in children far more rapidly and greater than hemodynamic responses at two minutes after the stimulus (Tables 2, 3). The pupillary responses to painful stimuli are far greater and more sensitive than the associated hemodynamic changes in children anesthetized with sevoflurane and 50% nitrous oxide. Constant et al. [1], in their study, found a decrease in pupil diameter two minutes after the injection of alfentanil and a return to pre-incision value after two minutes. Alfentanil is a good choice for fast study as its administration causes a rapid decline in pain-induced pupillary dilatation.

Saboudrin et al. [3] demonstrated the reduced requirement of intraoperative remifentanil in their study through the use of pupillometry during major gynecological surgery, which aligns with our findings. The authors reported the challenges in determining the appropriate threshold of pupil dilatation to assess analgesia.

Wildemeersch et al. [4] and Vide et al. [5], in their studies, have shown the efficacy of the use of PPI during general anesthesia with remifentanil. PPI shows the association of nociceptive surgical stimulus with pupillary reflex dilatation and a score of less than 4 was found to be associated with inhibition of nociceptive stimulus response. Sabourdin et al. demonstrated a decline in PPI score after a single dose of alfentanil, thereby analyzing nociceptive/antinociceptive response to the increase of nociception [6]. They concluded that PPI reflects the level of analgesia in children older than two years. We enrolled patients aged above two years (Table 1) in our study as per their study and other studies reflecting minimal change in pupil size in children below two years.

Boselli et al. [7] included 200 patients anesthetized with a halogenated agent or with propofol/remifentanil "at the discretion of the anesthesiologist". They demonstrated that the application of the nociceptive index for moderate-to-severe pain was also suitable for patients induced with a halogenated anesthetic. Senthil et al. [8] concluded that pupillometry is an easy-to-use, noninvasive bedside technique to measure nociception and monitor the effects of opioids. They recommended it as a utility tool for individualizing pain management in perioperative and ICU settings. In a few studies, pupil dilatation is compared with a verbal pain scale, and they have found it to correlate with pain intensity (VAS >4) in the postoperative period.

Julien-Marsollier et al. [9] assessed the diagnostic value of monitoring the ANI to detect surgical stimulation in children and found it to be an important surgical stimulation index. Wildemeersch et al. [10] showed that during propofol anesthesia, pupillometry with the possibility of low-intensity standardized noxious stimulation through PPI protocol can be used for the assessment of pupillary dilatation in response to the administration of remifentanil. When isoflurane concentration was increased from 0.7 to 1.0 and desflurane from 0.6 to 1.1 MAC, pupillary reflex dilatation did not increase. In our study, a surgical painful stimulus was applied at sevoflurane 1.5 MAC and N_2_O:O_2_ (50:50) to allow hemodynamic response in the range of the depth of anesthesia, and the applied MAC was above the MAC for surgical stimulus.

The conventional way to assess pain during anesthesia is through heart rate and blood pressure changes, which are neither specific nor sensitive to intraoperative pain. But it depends on various other factors like bleeding, respiration, the position of the patient, volume status, cardiovascular drugs used, and body temperature. No changes in terms of these parameters were seen in children in our study on surgical stimulus and only four (5.5%) showed an increase in heart rate >20% after one minute of surgical stimulus. However, pupillary dilatation was seen in almost all patients on painful stimulus, and it was significant after the stimulus and after opioid use (Tables 2, 3). Thus, pupil dilatation is a very sensitive and faster index for nociception than hemodynamic changes. Similar to our study, Larson et al. [11] measured the changes in pupil size after an electrical stimulus was applied to the abdominal skin in adults anesthetized with isoflurane or propofol and demonstrated that an intense noxious stimulus dilated the pupil by approximately 200% in less than one minute, the maximum rate depending on the hypnotic dose.

Opioids cause miosis in a dose-dependent manner, whose mechanism of action is on the parasympathetic nervous system [12]. Hence, we administered opioids when pupil dilatation was more than 13% of the baseline [13] at an incremental dose of 1 ug/kg. Inj. paracetamol 10 mg/kg was administered as rescue analgesic. The intraoperative consumption of opioids was found to be less than commonly prescribed.

In their study, Kegels et al. [13] included 20 patients scheduled for elective surgery, and pupillary reflex dilatation was observed by applying 10 mA automatic stimulation at an incremental dose of up to 60 mA, and stimulation stopped automatically when pupil size was >13%. Pupil size was measured twice with sevoflurane and with or without opioids. They reported no significant change in vital signs on noxious stimulation. The amplitude of dilatation and variation of pupil size decreased after opioid administration in both groups. This is similar to our study where administration of opioids decreased reflex pupil dilatation, causing pupil constriction, and hence the need for opioids for surgical stimulus, and guiding the use of opioids results in decreased consumption. The average duration of anesthesia was 1.4 ±0.5 hours, and hence there was almost no need for supplemental opioids intraoperatively; only 12 (16.6%) patients required analgesics in the postoperative period (Tables 4, 5). The incidence of side effects was minimal. This intervention carries a risk of ocular injury, and hence a conventional eyelid retractor was applied by the ophthalmologist (one used for ocular surgery), and precautions were taken to ensure that the measuring vernier caliper should not touch the pupil. An antibiotic eye drop was instilled in both eyes after the completion of surgery and at night.

Emery et al. [14] found greater pupillary dilatation in response to a noxious stimulus with tetanic stimulation in children aged above two years when testing the pupillary dilatation reflex as a method of estimating the sensory level in children receiving combined isoflurane/caudal epidural anesthesia when compared with children less than two years of age. Based on this, we selected children above two years of age in our study. Vinclair et al. [15],^ ^in their research, have shown the PPI score to be a predictive marker of pain response in sedated critically ill adults. Moreover, it has been proven that the administration of remifentanil reduces PPI scores. Our findings are similar to those of past research that shows that commonly monitored variables for analgesia like HR, SBP, and movement are less sensitive than PRD in adults as well as in children [15]

Our study included a substantial number (n=72) of pediatric patients as it was an invasive procedure, but it has its limitations too. The sample size can be increased for better results with the use of noninvasive devices for pupil size measurement. Since we did not have an automated infrared pupillometer at our institute, the measurement may not have been very accurate. The variables of pain assessment like heart rate and blood pressure are dependent on other factors too, such as age, volume status of the patients, and depth of anesthesia. Given these facts, we maintained the volume status of the children as per protocol and MAC 1.5 of sevoflurane was maintained during the measurement of pupil size. The light in the operation theater was kept ambient so that no bright light fell directly on the measuring eye.

## Conclusions

Based on our findings, pain has a significant influence on the pupil dilatation reflex in anesthetized children, and opioid administration based on pupil diameter can be of value in clinical settings. Although many scales like the visual analog scale and numeric rating scales are used to identify and measure pain in the postoperative area in conscious and awake patients, it is difficult to measure it perioperatively. Some other modalities such as evoked potential, brain imaging, EEG, or BIS can be helpful in monitoring pain and depth of anesthesia, but they are not easily available at all centers and need qualified personnel to operate them. An automated infrared pupillometer is a device to measure pupil diameter more accurately. The pupil dilatation reflex can assess pain, albeit in a controlled environment and free of confounding variables such as bright light and the use of some drugs.

The assessment of analgesia through pupil dilatation has definitive advantages over other autonomic responses to pain like heart rate and blood pressure, which are neither sensitive nor specific. Based on the above findings, we recommend that pupillary reflex dilatation be used to predict intraoperative pain and the requirement for opioids in anesthetized children. Further comparative studies are required to gain deeper insights into its sensitivity in predicting intraoperative pain and validate our results.
